# Placenta Prævia

**Published:** 1868-07

**Authors:** F. K. Bailey

**Affiliations:** Late of Joliet, Ill., now of Knoxville, Tenn.


ARTICLE XXIV.

### PLACENTA PRÆVIA.

By F. K. BAILEY, M.D., late of Joliet, Ill., now of Knoxville, Tenn.

During a practice extending over a period of 30 years, I have met with three cases of placenta prævia.

CASE I. June 2d, 1849 (Saturday), was called, towards night, to visit Mrs. D., living four miles from my residence; aged 20; first pregnancy; small in stature, and feeble in constitution. Found that she had slight uterine hemorrhage, and a little escape of liquor amnii, but not the least pain. She was "comfortable," pulse rather feeble, and complained of dizziness on sitting up. On examination per vaginam, found the os uteri undilated—could not reach so as to determine the presentation. There being no occasion for interference, I retired, leaving directions that I should be called, should any change occur. At

4 o'clock the next morning, made an examination, and found the condition as on the night before. There had been no pain during the night, and my patient could sit in a chair, or walk about the room. I then went home, but at 12½ o'clock was recalled, and arrived within an hour.

Found that at 11½ o'clock there was an instantaneous gush of blood from the vagina, which completely flooded the bed and ran through upon the floor. After the first flow had subsided, the hemorrhage was checked by means of cold applications made by the attendants. On my arrival, her face was pale as though she was dead; pulse feeble and quick; and the respiration very frequent and faint.

On examination, found the placenta mostly in the vagina, and one arm, with a loop of the cord, presenting at the os uteri. During all this, not the least pain had been complained of, and the os was dilated only about two inches, but smooth and rigid. Determining immediately to resort to turning, I found it very difficult to introduce the hand so as to seize the feet, by reason of a spasmodic contraction of the uterus upon its contents. After persevering effort version was at length effected, but still no expulsive pains were felt. There was no further loss of blood, for the probable reason that nearly all the vital fluid had already escaped.

Feeling confident that traction would endanger the safety of the patient, unless there should be expulsive action of the uterus, I gave ergot, with alcoholic stimuli; but in a few moments sinking commenced, which caused death in an hour.

I could not ascertain that there had been pain of any severity to cause the separation of the placenta before my arrival, and as it was almost wholly in the vagina when the examination was made, the fatal hemorrhage must have occurred in a few minutes after it commenced. What seemed peculiar, is the fact that there was no pain at any stage of the case, or, at least, but very slight.

Case II. The second case occurred March 4th, 1850, and the following is the record made of it at the time:

Mrs. W., aged 22, sanguine, nervous temperament, first pregnancy; taken about noon on the 3d inst. with slight uterine hemorrhage, and little or no pain. Flowing continued during the night, and some pain commenced towards morning. At 8 A.M., 4th, pain increased, as also did the hemorrhage. I first saw her at 9 A.M., and found her with a flushed face; pulse full, and 120 in a minute; slight uterine effort, attended with loss of blood at every pain. On examination, found the vagina filled with coagulated blood, and the os uteri but slightly dilated, but yielding. Felt a substance within the os like the edge of the placenta. The mouth of the womb was so high in the pelvis that it was difficult to ascertain, with any degree of certainty, the true nature of the case. The pains, however, were so slight that they were not at all expulsive, and, still, sufficient to cause hemorrhage.

With a view of quieting this feeble action which only caused wasting of the vital fluid, I gave $1\frac{1}{2}$ gr. opium. At 11 o'clock fainting occurred, upon which brandy and water was given, with another dose of opium, and towels wet in cold water applied to the uterine region. After about noon the pain and hemorrhage both nearly ceased; the extremities, which had become cold, began to be warm, and the patient was comparatively easy. Towards dark, expulsive pains came on, but with little or no flooding, and, on making an examination, I could distinctly feel the edge of the placenta, and, besides, the head pressing down, which pushed a "bag of waters." The membranes I at once ruptured, when the head immediately commenced descending and pressed upon the loosened edge of the afterbirth. From this time, expulsive efforts were violent and unattended with hemorrhage. About 9 o'clock, she was delivered of a stillborn child, weighing about 7 pounds.

The placenta was attached to the anterior portion of the cervix. The danger in this case seemed to be from loss of blood at each pain, however slight. When the membranes were ruptured, and the contents of the uterus brought to press upon the edge of the placenta, the bleeding vessels were closed. Had the feet or knees presented, fatal hemorrhage might have resulted before an adequate amount of pressure would have been made. I will add that the patient recovered as rapidly as could be expected, after losing such a quantity of blood.

Case III. The third case occurred but a few days ago, the history of which is as follows:

I was called March 2d, 1868, to visit Mrs. O., aged 20, good constitution, nervous, sanguine temperament. Found her complaining of severe pain in the left iliac region, and extending to the pubis, attended with copious uterine hemorrhage. On examination, found the os uteri slightly relaxed, but high in the pelvis; the cervix not entirely obliterated, and the patient thinks she is not more than 7½ months advanced. I suspected placenta prævia, and, to relieve the unnatural pain, gave a full dose of morphine, and ordered cold water applied to the uterine region.

*March 3d.* Found my patient relieved from pain, and that but little flowing had occurred since yesterday. Enjoined rest, and that I should immediately be called should any unfavorable symptoms arise.

*March 6th.* Was called, and found her in pain, as at first, and flowing copiously. Condition of the os unchanged. Prescribed opiates, cold applications, and rest.

*March 7th.* Pain relieved and no more hemorrhage.

*March 20th.* Was called about noon, and found her suffering from pain, attended with expulsion of large coagula. Os not dilated, and could not ascertain with certainty the presentation. Gave opium, which stopped both pain and flowing in a short time. At midnight was called, and found the woman had lost a great amount of blood. Syncope had resulted. The os was dilated about one inch, and I thought I could feel an edge of the placenta upon the posterior portion of the cervix. Gave stimulants, which only caused more pain and loss of blood. Each expulsive effort made it more evident that my diagnosis was correct, as an edge of the placenta could unmistakably be felt, and a firm coagulum between it and the uterus. At this juncture I ruptured the membranes, which had begun to protrude, and a copious flow of waters followed. Upon this, the head began immediately to descend and to press upon the loosened edge of the placenta. Within half an hour the hemorrhage entirely stopped, and labor was completed before daylight. The child, a male, was feeble at first, but soon breathed strongly. The woman is now doing well, although debilitated from loss of blood.

